# Logistic Bayesian LASSO for detecting association combining family and case-control data

**DOI:** 10.1186/s12919-018-0139-4

**Published:** 2018-09-17

**Authors:** Xiaofei Zhou, Meng Wang, Han Zhang, William C. L. Stewart, Shili Lin

**Affiliations:** 1 Department of Statistics, The Ohio State University, 1958 Neil Avenue, Columbus, OH 43210 USA; 2 Battelle Center for Mathematical Medicine, Nationwide Children’s Hospital Research Institute, 700 Childrens Drive, Columbus, OH 43205 USA

## Abstract

Because of the limited information from the GAW20 samples when only case-control or trio data are considered, we propose eLBL, an extension of the Logistic Bayesian LASSO (least absolute shrinkage and selection operator) methodology so that both types of data can be analyzed jointly in the hope of obtaining an increased statistical power, especially for detecting association between rare haplotypes and complex diseases. The methodology is further extended to account for familial correlation among the case-control individuals and the trios. A 2-step analysis strategy was taken to first perform a genome-wise single single-nucleotide polymorphism (SNP) search using the Monte Carlo pedigree disequilibrium test (MCPDT) to determine interesting regions for the Adult Treatment Panel (ATP) binary trait. Then eLBL was applied to haplotype blocks covering the flagged SNPs in Step 1. Several significantly associated haplotypes were identified; most are in blocks contained in protein coding genes that appear to be relevant for metabolic syndrome. The results are further substantiated with a Type I error study and by an additional analysis using the triglyceride measurements directly as a quantitative trait.

## Background

As next-generation sequencing (NGS) technology becomes more accurate and affordable, many recent studies have focused on assessing associations between common complex diseases and single-nucleotide variants (SNVs), paying particular attentions to those that are rare. Various methods have been proposed, but most can only achieve the identification of candidate genes or regions. To narrow the list of potential causal variants, it would be helpful to investigate haplotype blocks formed by single-nucleotide polymorphisms (SNPs) in regions/genes where associations are suggested but may not necessarily be genome-wide significant. Apart from being able to identify biologically relevant variants, haplotype-based methods can be more powerful than SNV-based methods as multilocus genotypes contain more information than single-locus genotypes, especially when causal loci interact in *cis*, leading to disease etiology [1]. If there are rare causal SNVs in a haplotype block, then rare haplotypes can tag such causal variants, a conclusion based on a simulation study [2]. More importantly, rare haplotypes may be obtained from common SNPs, rendering NGS data unnecessary. The power for detecting rare haplotype associations is further enhanced in a family-based study, as rare associated variants are enriched in families afflicted with the diseases compared to population samples of independent cases and controls of the same size. Currently, numerous methods exist, including a class based on Logistic Bayesian LASSO (LBL) for detecting associations of haplotypes, common or rare, using either case control or family-based data [1, 3].

The GAW20 Real Data Package provides a good opportunity to apply LBL to identify haplotypes that are associated with metabolic syndrome. Specifically, we consider the ATP binary trait derived from the measurements taken at visit 2 (before drug intervention). Among the 188 pedigrees in the data set, only 17 contain complete case–parent trios (ie, genotype information for both parents and the child and phenotype status for the child are all available), leading to a total of only 25 such trios. In addition, we extracted 283 cases (ATP = 1) and 475 controls (ATP = 0) with available genotype information. Because the number of trios is extremely small, it is clear that there is insufficient power to detect haplotype association using these data alone. However, their inclusion may enhance detection power compared to when only case-control data are used. Because the current LBL methodology focuses on a single study design, we propose eLBL, an extension of the LBL methodology to combine case–control and case–parent trios data for a joint analysis. Furthermore, because cases, controls, and trios are all extracted from the same set of pedigrees, there are intrinsic correlations. To account for such familial dependency, we have adopted a composite likelihood adjustment approach.

## Methods

### Extension of the logistic Bayesian LASSO accounting for familial dependency

Suppose we have *n* = *n*_1_ + *n*_2_ individuals, where *n*_1_ and *n*_2_ are the numbers of cases and controls, respectively. Let *Y* = (*Y*_1_, *Y*_2_,...,*Y*_*n*_), where *Y*_*i*_ denotes the affection status of the *i*th individual, with case = 1 and control = 0. Let *Z* = (*Z*_1_, *Z*_2_,...,*Z*_*n*_), where *Z*_*i*_ is the (unobserved haplotype pair) of individual *i*, while the observed genotype matrix is denoted by *G* = (*G*_1_, *G*_2_,...,*G*_*n*_), where *G*_*i*_ is the genotype vector (over a set of SNPs) of the *i*th individual. We note that *G* contains information about *Z*, but the mapping is typically many (haplotype pairs) to one (vector of genotypes). The complete data (haplotype) likelihood is

1 $$ {L}_c\left(\varnothing \right)={\prod}_{i=1}^{n_1}P\left({Z}_i|{Y}_i=1,\varnothing \right){\prod}_{j={n}_1+1}^nP\left({Z}_j|{Y}_j=0,\varnothing \right) $$

where the probabilities are specified, as elaborated below, through a logit link function relating the odds of disease to the haplotypes, and ∅ is a vector of parameters including haplotype frequencies and coefficients of the logistic regression model.

Suppose we also have *m* trios with each ascertained through the offspring. Let *Y*_*ic*_ = 1 denote that the child is a case. The haplotype configuration of the *i*th trio can be written as *Z*_*i*_ = (*Z*_*if*_, *Z*_*im*_, *Z*_*ic*_), where the 3 components denote the haplotype pair of the father, mother, and the affected child, respectively. Under the assumption of allelic exchangeability, this is equivalent to *Z*_*i*_ = (*Z*_*ic*_, *Z*_*iu*_), where *Z*_*iu*_ is the untransmitted haplotype pair from the parents. Then, the haplotype-based likelihood for case–parent trios is

2 $$ {L}_f\left(\varnothing \right)={\prod}_{i=1}^mP\left({Z}_{ic}|{Y}_{ic}=1,\varnothing \right)P\left({Z}_{iu}|\varnothing \right) $$

where the probabilities and the parameter vector are specified as in the case–control data. Putting eqs. (1) and (2) together, we obtained the following composite likelihood:

3 $$ {L}_{cf}\left(\varnothing \right)={L}_c\left(\varnothing \right)\times {L}_f\left(\varnothing \right) $$

It is apparent from the description of the GAW20 data given above that our data units are not independent, thus the composite likelihood as specified in eq. (3) is not the correct likelihood based on the observed data. However, owing to the complex relationships among the extracted cases, controls, and trios, it is difficult to formulate the correct likelihood. Fortunately, it is possible to obtain correct inferences based on the misspecified composite likelihood *L*_*cf*_(∅) through appropriate adjustment [4]. Following the “magnitude adjustment” algorithm [5], we denote *H*(∅) =  − *E*[*∇*^2^*ℓ*_*cf*_(∅)]and *J*(∅) = *Var*[*∇ℓ*_*cf*_(∅)], where *ℓ*_*cf*_(∅)=log[*L*_*cf*_(∅)] is the log-composite likelihood, and ∇ and ∇^2^ are the first-order and second-order derivatives, respectively. Let *λ*_1_, *λ*_2_, ⋯, *λ*_*p*_be the eigenvalues of *H*(∅)^−1^*J*(∅); based on them we form$k=p/{\sum}_{i=1}^p{\lambda}_i.$ Then the adjusted log-likelihood

4 $$ {\ell}^{\ast}\left(\varnothing \right)={k\mathit{\ell}}_{cf}\left(\varnothing \right) $$

is used for inference, as elaborated in the following paragraph.

To specify the probabilities and elaborate on ∅, we assume that, for any given individual in the study with haplotype pair *Z*, we model the odds of the disease *θ*_*Z*_ = *P*(*Y* = 1|*Z*)/*P*(*Y* = 0|*Z*) with a logistic model log*θ*_*z*_ = *α* + *X*_*Z*_*β*, where X_Z_ is the design vector corresponding to haplotype pair *Z*, coded according to the assumed mode of inheritance (eg, additive, recessive, dominant); *β* = (*β*_1_, ⋯, *β*_*K*_) (part of the collection of parameter vector ∅) is the regression coefficient vector with *β*_*j*_ corresponding to the effect of the *j*th variant on the log odds; and α is the baseline effect (related to the phenocopy rate). Note that if we assume an additive model, then the *j*th variant is the *j*th haplotype, and the total number of distinct haplotypes is *K* + 1. We cast the problem into a Bayesian framework, where the adjusted likelihood in eq. (4) is used for correct posterior inference [5]. The detailed Markov chain Monte Carlo (MCMC) inference procedure follows the original LBL methodology using shrinkage priors to increase power for detecting rare haplotypes [1, 3]; the adjustment factor *k* is updated in each MCMC iteration. Convergence of the Markov chain is assured based on commonly used diagnostic tools. The posterior odds over the prior odds, namely the Bayes factor (BF), is used to assess the significance of the *β*_*j*_s. We have also constructed empirical posterior credible intervals (CIs) for the odds ratios (ORs). For each haplotype, the OR is essentially the exponential of the corresponding *β* in the logistic model given above. It is estimated, together with the CIs, from the posterior sample of the *β* values. Decision on the significance of a haplotype is based on both BF (> 2) and CI (not including the null value 1).

### A 2-step analysis strategy

Because the proposed eLBL (extension of the Logistic Bayesian LASSO [least absolute shrinkage and selection operator]) methodology is based on an MCMC procedure to sample from the posterior distribution, it is computationally intensive, and thus not suitable for whole-genome scan. Instead, we adopt the following 2-step strategy. In the first step, we use Monte Carlo pedigree disequilibrium test (MCPDT) [6], a family-based single-SNP association testing method, to scan 654,767 SNPs across the 22 autosomes. We excluded SNPs with low minor allele frequencies (< 1%). MCPDT imputes missing data and takes familial relationships into account; consequently, it is viewed as using all information to the maximum extent possible. In the second step, we formed haplotype blocks around the SNPs selected from Step 1 using haploview [7]. We then applied eLBL to identify haplotype(s) within each block that have a significant influence on the ATP binary trait.

## Results

Of the 654,767 SNPs considered, we selected the 10 SNPs with the smallest MCPDT *p* values for further analysis with eLBL. These 10 SNPs have *p* values close to 10^− 4^ or smaller (Table 1), with the 3 SNPs on chromosome 1 passing the threshold of genome-wide significance at the 5% level after Bonferroni correction. To increase the statistical power for detecting rare variants and to potentially understand the causal mechanism in downstream analysis, we applied eLBL to 9 haplotype blocks that cover the SNPs displayed in Table 1. Note that the first 2 SNPs on chromosome 1 belong to the same haplotype block (block 1; Table 1). The remaining 8 SNPs were placed into 8 separate blocks labeled as blocks 2 to 9 (Table 1).

**Table 1:** Top 10 SNPs with the smallest *p* values as identified by MCPDT

SNP	Chr	Position	Allele^a^	MAF	*P v*alue	Block
rs10915052	1	30,479,266	G/A	0.0104	2*.*59 *×* 10^*−* 8^	1
rs1406862	1	30,483,442	T/C	0.0109	3*.*48 *×* 10^*−*8^	1
rs16833496	1	30,509,960	G/A	0.0110	1*.*80 *×* 10^*−*7^	2
rs17086804	4	57,033,788	T/C	0.0597	1*.*22 *×* 10^*−*4^	3
rs2048091	8	19,003,646	A/C	0.3565	5*.*92 *×* 10^*−*5^	4
rs12281650	11	69,360,392	C/T	0.2665	9*.*48 *×* 10^*−*5^	5
rs7943255	11	124,450,756	G/A	0.0544	1*.*23 *×* 10^*−*4^	6
rs8001893	13	33,120,261	C/A	0.0171	6*.*24 *×* 10^*−*6^	7
rs35625	16	16,077,067	T/C	0.4123	1*.*15 *×* 10^*−*4^	8
rs966287	18	27,580,619	G/C	0.3285	8*.*95 *×* 10^*−*5^	9

MAF, minor allele frequency

^a^Minor allele is listed after the slash

The results from eLBL, presented in Table 2, show that a number of haplotypes have an estimated CI of OR not including 1 and a BF > 2, providing significant evidence for association between the identified haplotypes and the ATP trait. For instance, haplotype h11110 of block 1, which contains the minor alleles of SNPs rs10915052 and rs1406862 (occupying the first and fourth positions, in bold in Table 2), is seen to have a fairly significant evidence of association with a BF of 15. That this haplotype contains the 2 minor alleles strongly suggests that the 2 SNPs may very well interact in *cis* and play a regulatory role for metabolic syndrome, as the block is not located within the coding region of a gene. As another example, 2 haplotypes in block 7, located within the protein coding gene *STARD13*, are inferred to be associated with ATP. This haplotype block spans 10 SNPs, with SNP rs8001893 sitting at the last position. For the haplotype containing the minor allele, h1111111111, its effect is protective (OR < 1), which is opposite of the effect of haplotype h1000000000 (OR > 1). Both haplotypes are rare, especially the risk haplotype (frequency < 0.001). Given its rarity and the large variability in the estimate (reflected in the large upper bound of the CI resulting from small frequency and only moderate sample size), care needs to be taken in the interpretation of the effect size. Nevertheless, the associated gene appears to be relevant for the study of metabolic syndrome. Gene Ontology annotations related to *STARD13* include guanosine triphosphatase (GTPase) activator activity and lipid binding. Finally, another protein coding gene, *ABCC1*, which contains haplotype block 8, is also noteworthy, as it also appears to be pertinent to metabolic syndrome. Among its related pathways are vitamin digestion and absorption, and metabolism.

**Table 2:** Significant haplotypes identified by eLBL; CI does not include 1 and BF > 2

Block^a^	Chr	Gene	Hap^b^	Freq	OR	LB^c^	UB^c^	BF
B1	1	NA	h**1**11**1**0	0.0103	0.2167	0.0482	0.7293	14.88
B3	4	*SRP72*	h00**1**10011	0.0601	0.5195	0.3137	0.8374	9.66
B4	8	*ncRNA*	h0**1**000	0.1494	1.6192	1.1460	2.3157	7.36
B6	11	*SLC37A2*	h**1**11000	0.0361	0.3751	0.1651	0.7630	18.64
B7	13	*STARD13*	h100000000**0**	0.0006	77.9239	1.7253	3085.8835	20.70
			h111111111**1**	0.0164	0.2731	0.0814	0.7502	15.32
B8	16	*ABCC1*	h0**1**01010	0.1663	0.7062	0.5272	0.9435	2.09

^a^The SNPs contained in the haplotype blocks are as follows: B1: rs10915052 rs2377270 rs2205841 rs1406862 rs12410878; B3: rs6849183 rs11133443 rs17086804 rs11610 rs41476944 rs17086853 rs12649799 rs10015634 B4: rs10104096 rs2048091 rs13266438 rs10088192 rs13262422; B6: rs7943255 rs12289510 rs12276567 rs10893317 rs4936976 rs3808995; B7: rs9563616 rs7985396 rs9591912 rs8001801 rs7993044 rs9315232 rs10507413 rs9569943 rs7328696 rs8001893; B8: rs35621 rs35625 rs4148350 rs4148351 rs35628 rs4148353 rs35629

^b^“1” denotes the minor allele and the SNPs in Table 1 are in bold

^c^LB (lower bounds) and UB (upper bounds) of the odds ratio (OR), which make up the 95% credible interval (CI)

## Discussion and conclusions

Motivated by making maximum use of information resulting from the limited sample sizes when only case–control or trio data are considered, we propose eLBL, an extension of the LBL methodology, so that both types of data can be analyzed jointly to increase statistical power. This new approach is further extended to adjust for familial correlations, leading to correct statistical inference using dependent data. Our 2-step analysis strategy was designed to increase statistical power. Indeed, by using all available information, MCPDT identified 3 genome-wide significant SNPs, which disappear when only observed data are used (results not shown). On the other hand, eLBL with shrinkage priors was able to recover haplotypes (many are rare) that are associated with the ATP binary trait. The associated genes harboring the haplotype blocks studied all appear to be related to metabolic syndrome. The increase in power is clearly seen as several of the associated haplotypes contain SNPs that do not pass genome-wide significance. To further substantiate the gain in power with the new eLBL approach, we performed an analysis with only independent cases and controls using the original LBL [1]. The results, as expected, are sensitive to the selection of the independent samples, and miss many of the haplotypes identified in Table 2. Similarly, an analysis of 17 independent trios using famLBL [2] reveals that the sample size is too small to obtain interpretable results. With an increase in power, the natural question is whether there is also an increase in Type I error, as eLBL is a new method and has not been studied thoroughly. To answer this question, we performed a limited simulation study wherein data from a null model was simulated. To mimic the family dependent structure and the linkage disequilibrium structure of the real data, we simulated our data using the GAW20 families and the inferred haplotypes with the estimated frequencies from block 8 to preserve linkage disequilibrium. Our results indicate that there is no elevated Type I error. In the contrary, eLBL is seen to be conservative for rare variants. Nevertheless, for haplotypes with frequencies greater than 0.05, the Type I error is as expected. To further substantiate the results from eLBL, we also analyzed the triglyceride level from visit 2 directly as a quantitative trait using a variation of LBL [1], but also accounting for the familial structures in the data. Of the 4 protein coding genes, *SRPT2*, *SLC37A2*, *STARD13*, and *ABBC1*, identified by eLBL, the quantitative analysis also identified associated haplotypes in blocks contained within these genes. These results, together with the Type I error study and the annotations of the genes, affirm the results from eLBL, leading to our conjecture that the haplotypes identified are potentially either involved in the causal mechanism or playing a regulatory role in metabolic syndrome.
